# The Different Characteristics of Cirrus Optical Coherence Tomography between Superior Segmental Optic Hypoplasia and Normal Tension Glaucoma with Superior Retinal Nerve Fiber Defect

**DOI:** 10.1155/2015/641204

**Published:** 2015-05-13

**Authors:** Jong Chul Han, Da Ye Choi, Changwon Kee

**Affiliations:** Department of Ophthalmology, Samsung Medical Center, Sungkyunkwan University School of Medicine, Seoul 135-710, Republic of Korea

## Abstract

*Purpose*. To evaluate the different characteristics in superior segmental optic hypoplasia (SSOH) and normal tension glaucoma (NTG) with superior retinal nerve fiber layer (RNFL) defect (NTG-SRD) compared to normal control using cirrus optical coherence tomography (OCT). *Methods*. SSOH eyes and NTG-SRD eyes were reviewed. The peripapillary RNFL (pRNFL) and ganglion cell inner plexiform layer (GCIPL) of the two groups were compared to age-matched normal controls using cirrus OCT. *Results*. Included in this study were 31 SSOH eyes, 33 NTG patients, and 49 healthy normal controls. Compared to normal controls, pRNFL thickness in SSOH eyes was thinner except in the inferotemporal to the temporal segment. NTG-SRD eyes had thinner pRNFL except in the nasal to inferonasal segment. Meanwhile, GCIPL thickness in SSOH eyes was thinner in the global and sectoral segment, but not in the superonasal and inferonasal sectors compared to normal controls. NTG-SRD eyes showed thinner GCIPL in all sectors compared to normal controls. In case of comparison between SSOH and NTG-SRD, superonasal sector was thinner in NTG-SRD than in SSOH (*P* = 0.03). *Conclusions*. The different distributions of nerve fiber layer were shown in pRNFL and GCIPL between SSOH eyes and NTG-SRD eyes.

## 1. Introduction 

Superior segmental optic hypoplasia (SSOH) is a developmental anomaly characterized by a relative hypoplasia of the superior portion of the optic nerve head and retinal nerve fiber layer [1, 2]. The characteristic findings of SSOH include a relatively superior entrance of the central retinal artery, thinning of the superior retinal nerve fiber layer (RNFL), a superior peripapillary scleral halo, and superior optic disc pallor. SSOH is also characterized by visual field defects such as inferior altitudinal or sector-like field defect and normal visual acuity [3–5].

The prevalence of SSOH was reported 0.24 to 0.3% in the Asian population [1, 2]. Although much is known about the clinical presentation of SSOH, the pathogenesis of SSOH remains uncertain. SSOH is generally regarded as a nonprogressing congenital disorder. Therefore, it is important to differentiate SSOH from glaucoma because SSOH patients do not need a treatment. However, it is not easy to differentiate the two diseases because SSOH and glaucoma have similar features, such as localized RNFL thinning and neuroretinal rim thinning [6]. Furthermore, the two diseases sometimes exist simultaneously [7, 8].

Recently, optical coherence tomography (OCT) has been used to better understand the optic nerve head structure in SSOH, as well as in glaucoma. Using OCT to assess the thickness of the peripapillary RNFL (pRNFL) and the ganglion cell inner plexiform layer (GCIPL) has been suggested to be additional diagnostic method for glaucoma [9, 10]. We hypothesized that GCIPL may show different distribution between the two groups and help to differentiate SSOH from normal tension glaucoma (NTG) especially with superior RNFL defect (NTG-SRD). The goal of this study was to investigate whether measurement of GCIPL using cirrus OCT is helpful to differentiate SSOH from NTG-SRD.

## 2. Materials and Methods

The present study was a case-control study approved by the Institutional Review Board of Samsung Medical Center and adhered to the tenets of the Declaration of Helsinki. The medical charts of SSOH and NTG patients who visited Samsung Medical Center from January 2012 to December 2013 were reviewed. We chose age-matched normal controls from a pool of subjects who were diagnosed to be “within normal limits” at the glaucoma clinic at Samsung Medical Center.

All patients and normal control subjects underwent routine ophthalmologic examinations including best-corrected visual acuity (BCVA), spherical equivalent (SE), slit-lamp biomicroscopy, Goldmann applanation tonometry (GAT), gonioscopy, optic disc photo, red-free RNFL, and visual field (VF) testing by the Humphrey Field Analyzer Model 750I (Humphrey Instruments Inc., San Leandro, CA, USA), using the program Central 30-2, SITA-standard strategy. The global indexes as mean deviation (MD), pattern standard deviation (PSD), and visual field index (VFI) were used for comparison of VF defect among the groups.

Cirrus OCT (Carl Zeiss Meditec, Inc., Dublin, CA) was used to assess the morphological characteristics in the optic nerve head regions (optic disc cube 200 × 200 protocol) and macula (macular cube 514 × 128 protocol) after pupil dilatation. Of note, the optic disc cube protocol was used for RNFL analysis and the macular cube protocol was used for ganglion cell layer analysis. Only good quality scans with a signal strength of 7 were used for the analysis. The mean, sectoral (temporal, superior, nasal, and inferior), and clock hour pRNFL thickness measurements were analyzed. The mean, minimum, and sectoral (superior, superonasal, inferonasal, inferior, inferotemporal, and superotemporal) GCIPL thickness measurements were analyzed. The optic disc area, among the parameters of ONH analysis, was used for the analysis.

SSOH patients had to meet the following criteria [6]: (1) the optic disc having the characteristic features of SSOH, at minimum, rim thinning of the optic nerve head most prominent in the superior nasal region; (2) RNFL thinning in the superior nasal region; (3) visual field (VF) testing revealing inferior arcuate or sector-like defects; (4) IOP being less than 22 mmHg by GAT; (5) no VF progression for at least three years. VF progression was defined as at least 3 test points exhibiting significant (*P* < 0.05) progression at the same location on 3 consecutive tests as compared with the baseline. NTG-SRD was defined as follows: (1) optic nerve head changes such as focal or generalized narrowing or notching of the superotemporal rim, disc hemorrhages; (2) RNFL defects correlating to glaucomatous changes of the optic nerve head; (3) glaucomatous VF defects that showed at least two of the following criteria and should exist in more than one reliable test: (1) a cluster of three points with probability of less than 5% on the pattern deviation map in at least one hemifield and including at least one point with a probability of less than 1% or a cluster of two points with a probability of less than 1%; (2) glaucoma hemifield test results outside normal limits; (3) a pattern standard deviation of 95% outside the normal limits; or (4) an early glaucomatous VF defects above −6 dB of MD value; (4) IOP less than 22 mmHg by GAT; (5) glaucomatous VF progression that should be confirmed at least once during the follow-up period. The following cases were excluded: (1) previous retinal disease history; (2) previous ocular surgery or trauma history; (3) the presence of neurologic disease that could affect the visual field.

The ANOVA test and Tukey's post hoc analysis were used for comparisons of the pRNFL and GCIPL parameters among the SSOH eyes, NTG-SRD eyes, and the normal controls. Categorical variables were compared using chi-square test. Clock hour thickness values from the left eyes were converted into the right eye format. Statistical analyses were performed using SPSS 18.0 (SPSS Inc., Chicago, IL, USA). The results of ANOVA and Tukey's post hoc analysis were considered significant at *P* values < 0.05.

## 3. Results

**Figure 1:**
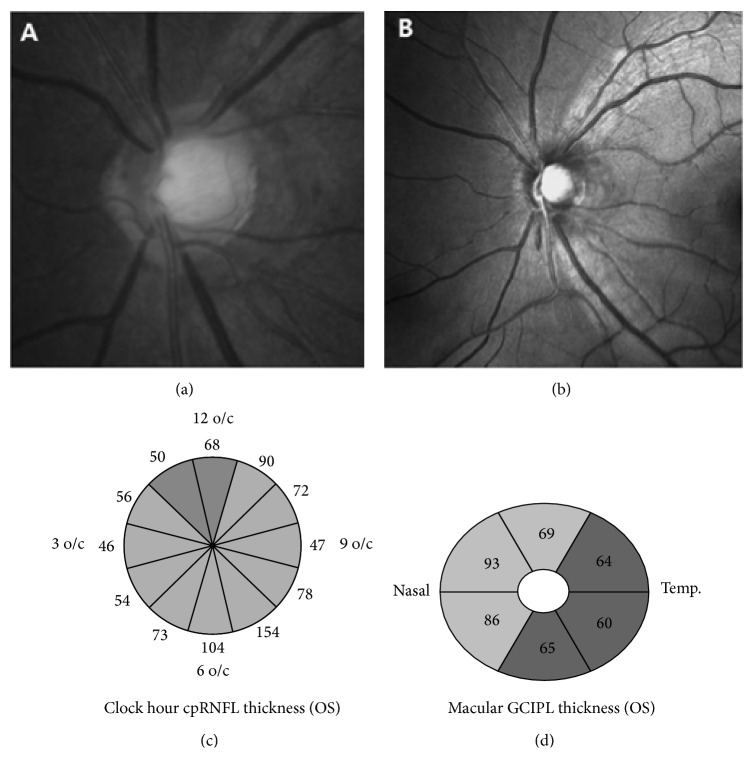
An example of an eye with superior segmental optic hypoplasia (SSOH) in 45-year-old female patient. (a, b) Color fundus photograph and red-free retinal nerve fiber layer (RNFL) photograph of the left eye show optic disc rim thinning and RNFL defect in the superior and superonasal segments. (c) Optical coherence tomography (OCT) shows definite thinning of RNFL in the superior and superonasal segments. (d) Macular analysis in OCT shows generalized thinning of ganglion cell inner plexiform layer (GCIPL) except superonasal and inferonasal sectors.

**Figure 2:**
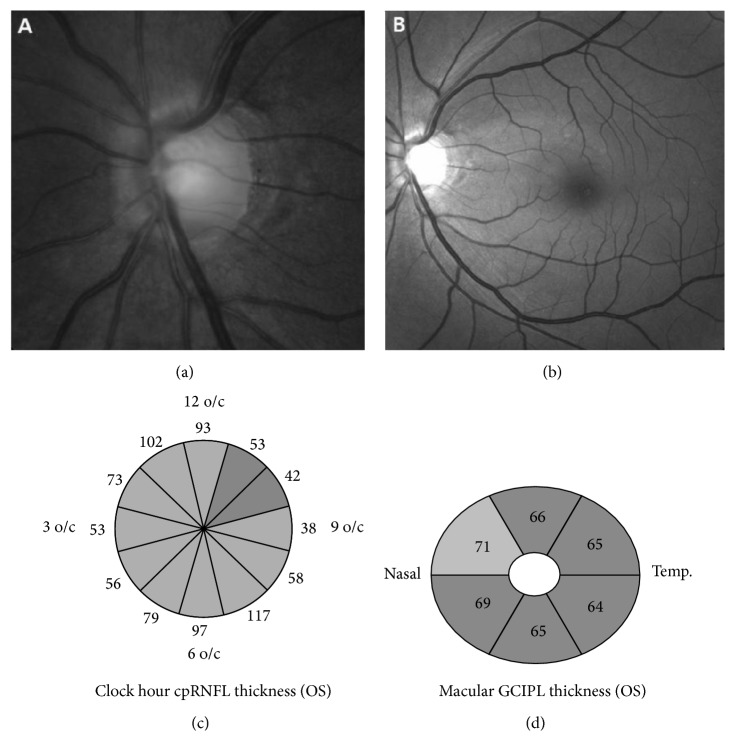
An example of an eye with normal tension glaucoma (NTG) with superior retinal nerve fiber layer (RNFL) defect in a 46-year-old male patient. (a, b) Color fundus photograph and red-free retinal nerve fiber layer (RNFL) photograph of the left eye show optic disc rim thinning and RNFL defect in the superotemporal segment. (c) Optical coherence tomography (OCT) shows definite thinning of RNFL in the superotemporal segment. (d) Macular analysis in OCT shows generalized thinning of ganglion cell inner plexiform layer (GCIPL) in all sectors.

The demographics and ocular characteristics of the 31 eyes of 31 SSOH subjects and the 33 eyes of 33 NTG patients and the 49 eyes of 49 healthy normal controls are presented in Table 1. The figures display the ophthalmologic findings via photos and OCT images in SSOH eyes (Figure 1) and in NTG-SRD eyes (Figure 2). The mean age was 44.9 ± 7.0 years in SSOH eyes, 47.3 ± 5.4 years in NTG-SRD eyes, and 44.8 ± 6.4 years in normal controls (*P* = 0.51). The male to female ratio was 13 : 18 in SSOH eyes, 15 : 18 in NTG-SRD eyes, and 17 : 32 in normal controls (*P* = 0.87). No differences were found in SE, BCVA, and IOP among groups (*P* = 0.41, *P* = 0.08, and *P* = 0.38) (Table 1). The average disc area was significantly smaller in SSOH eyes than in both NTG-SRD eyes and normal controls (1.7 ± 0.36 mm^2^ in SSOH, 1.94 ± 0.51 mm^2^ in NTG-SRD, and 1.95 ± 0.43 mm^2^ in normal controls; *P* = 0.01). The average values of MD, PSD, and VFI were statistically different among the groups, but there were no significant differences between SSOH eyes and NTG-SRD eyes (MD, *P* = 0.57; PSD, *P* = 0.12; VFI, *P* = 0.12) (Table 1).

In Table 2, the differences in the pRNFL thickness and GCIPL thickness among the groups are shown. SSOH eyes had thinner pRNFL than normal controls, except for at the 6 to 9 o'clock segment (inferotemporal to temporal segment), and NTG-SRD eyes showed thinner pRNFL, except for at the 2 to 6 o'clock segment (nasal to inferonasal segment). When comparing SSOH eyes to NTG-SRD eyes, the pRNFL thickness in the SSOH eyes at the 1 and 2 o'clock segment (superonasal segment) was significantly thinner than NTG-SRD eyes (*P* = 0.02; *P* = 0.04, resp.). However, the segment from 10 and 11 o'clock (superotemporal segment) was thinner in the NTG-SRD eyes than in the SSOH eyes (*P* = 0.01; *P* = 0.01, resp.). In the analysis of GCIPL, SSOH eyes had thinner GCIPL in both global and sectoral thickness measurements except for the superonasal and inferonasal sectors (*P* = 0.16; *P* = 0.20, resp.) compared to normal controls. NTG-SRD eyes showed thinner GCIPL in all sectors compared to normal controls. In case of comparison between SSOH eyes and NTG-SRD eyes, superonasal sector was thinner in NTG-SRD than in SSOH (*P* = 0.03) (Table 2).

## 4. Discussion 

Differential diagnosis between SSOH eyes and NTG-SRD eyes is sometimes difficult. Although the characteristic features of SSOH eyes have been discussed in previous studies, some of the characteristic features are rarely found in the Asian population [1, 11, 12]. Yamamoto et al. suggested that the clinical features of SSOH, such as disc pallor and a superior peripapillary scleral halo, may be found lesser in Japanese people than in Western population. Therefore, they suggested that thinning of the RNFL and ONH in the superior and superonasal segments with corresponding VF defects are essential for the diagnosis of SSOH [13]. In our previous study, only thinning in the superior and superonasal segments of the pRNFL, combined with rim thinning of the corresponding segments, was found to be the essential factor for the diagnosis [6]. At this point, it seems that detecting superonasal rim thinning, finding RNFL defect, and the presence of consistent inferior altitudinal or sector-like VF defect are the most reliable criteria to confirm the diagnosis of SSOH. In our study, the same criteria were chosen as previously reported in an Asian population [13] and the other characteristics such as superior entrance of the central retinal vessels, superior peripapillary halo, or superior optic disc pallor were suboptimal.

Recently, the diagnosis of SSOH has been supported by development of imaging devices, which allow more accurate and objective measures of disc morphology. According to Unoki et al. [12], using Stratus OCT to explore SSOH results in a TSNIT curve with a “single hump” pattern. In our previous report, we also showed the same pattern using cirrus OCT and we showed the greatest AUROC to be the 1 o'clock segment in the superonasal portion of the pRNFL [6]. Yamada et al. reported similar results, in that the RNFL of SSOH eyes were thinner in the superior to superonasal segment using Stratus OCT [14]. In the present study, SSOH eyes showed thinner pRNFL than normal controls except for the inferotemporal to temporal area compared to age-matching normal controls. Meanwhile, NTG-SRD eyes showed thinner pRNFL except for the nasal to inferonasal area compared to age-matching normal controls. It is interesting that thinner pRNFL than normal controls was found at inferonasal area in SSOH eyes and at inferotemporal area in NTG-SRD eyes though no significant RNFL defect on the area was found. When comparing SSOH eyes and NTG-SRD eyes, SSOH patients had thinner superonasal area (1 and 2 o'clock, resp.) and NTG-SRD patients had thinner superotemporal area (10 and 11 o'clock, resp.).

The availability of the GCIPL measurement in glaucoma has been known as follows [10, 15]. First, RNFL consists of retinal ganglion cell axons, and therefore evaluation of the RGCs may be a better method for measuring glaucomatous damage than pRNFL thickness. Furthermore, over 50% of RGCs are located in the macula and the scanning of macula means the overall screening of the entire RGCs in the retina [16]. Therefore, GCIPL was regarded as the alternative method to detect even slight RGC layer changes. In the present study, we found that GCIPL in SSOH eyes was thinner than normal controls except for the SN and IN sectors, whereas all GCIPL sectors in NTG-SRD eyes were thinner than normal controls. In case of comparison between SSOH eyes and NTG-SRD eyes, NTG-SRD eyes showed significantly thinner SN sector than SSOH eyes. We hypothesized that the results are due to the fact that the RNFL defect in NTG-SRD eyes was involved nearer to fovea than SSOH eyes. Hwang et al. suggested that GCIPL can offer good ability to detect early glaucoma when the angular distance between the fovea and the RNFL defect is small [17]. The vulnerable location of SSOH and NTG-SRD seems to be different, and this is the reason why the two diseases showed different RNFL distributions in pRNFL and GCIPL.

Meanwhile, SSOH eyes had a relatively smaller disc size than both NTG-SRD eyes and normal controls. Small disc size has been suggested as a risk factor of glaucoma associated with fewer optic nerve fibers and smaller anatomic reserve capacity though it is controversial [18, 19]. The point was made that SSOH eyes have a small disc size with thinner RNFL and GCIPL simultaneously, which suggests that SSOH patients may have been born with thin RNFL and a small number of RGCs, making them susceptible to glaucomatous changes. This idea of susceptibility is consistent with our previous report showing the high prevalence of glaucoma in SSOH [7].

Only patients with no VF progression were included in the SSOH eyes and patients with VF progression were included in the NTG-SRD during the follow-up period in the present study. Follow-up observation for detecting VF progression seems to be another essential point in differentiating SSOH from NTG-SRD. Because prevalence of glaucoma may be higher even in SSOH patients, thus persistent follow-up observation is needed even though the diagnosis is likely to be SSOH [7]. In the present study, several SSOH patients were excluded due to VF progression during follow-up period. Interestingly, in the excluded patients, the average SE was −5.1 D and the proportion of high myopia (<−6.0 D) was approximately 42.8%. Myopia is one of the risk factors of glaucoma [20, 21], but no previous reports suggested that high myopia may be associated with glaucomatous progression in SSOH eyes. In one previous case report of progressive SSOH, the patient had a high myopia of −8.0 D [8], so it might not be coincidence that glaucoma with SSOH had a high myopia, and it seems necessary to evaluate whether coexistence of SSOH and high myopia can lead to glaucomatous changes or progression.

This study has several limitations. First, this study is based on a small number of cases. Because the prevalence of SSOH is quite low, including an adequate number of patients was difficult. Second, some factors that can affect RNFL thickness and distribution were not excluded. These factors such as optic disc torsion or myopia can temporalize the RNFL peak, which could affect the results of this study. However, there were no differences in SE among SSOH, NTG-SRD, and normal controls and they showed mild myopia so we thought the influence of the potentially confounding factors on the results would be negligible. Third, the type II error could be increased by using the comparisons including normal eyes instead of direct comparison only between SSOH and NTG-SRD. However, this indirect method was used because we intended to show overall different RNFL distribution in SSOH that is a clinically rare disease. Fourth, the measurement bias should be considered because pRNFL and GCIPL could not be independent. Due to smooth change of thickness profile, it is likely for two nearby sectors to have a similar thickness of pRNFL and GCIPL. Though it is not certain how much the bias could affect the result, it is needed to consider this effect when we understand the present study result.

In conclusion, GCIPL showed thinner superonasal sector in NTG-SRD eyes than in SSOH eyes. In addition, pRNFL showed thinner superonasal segment in SSOH than in NTG-SRD and thinner superotemporal segment in NTG-SRD than in SSOH. The different characteristics of the pRNFL and GCIPL of SSOH and NTG-SRD eyes should be considered when the clinician diagnoses the two diseases that are clinically similar.

## Figures and Tables

**Table 1:** Demographics and ocular characteristics.

	SSOH (A)	NTG-SRD (B)	Controls (C)	*P* value	*P* value		
					Post hoc comparison		
					A-B	A-C	B-C
Eyes (*N*)	31	33	49				
Age (years)	44.9 ± 7.0	47.3 ± 5.4	44.8 ± 6.4	0.51			
Sex							
Male	13	15	17	0.87			
Female	18	18	32				
SE (D)	−1.9 ± 1.3	−1.7 ± 1.9	−1.3 ± 1.4	0.41			
BCVA	0.99 ± 0.03	0.92 ± 0.13	0.97 ± 0.08	0.08			
IOP (mmHg)	14.3 ± 2.0	15.0 ± 3.2	15.0 ± 2.9	0.38			
Disc area (mm^2^)	1.70 ± 0.36	1.94 ± 0.51	1.95 ± 0.43	0.01	0.004	0.003	0.92
Mean deviation, dB	−2.7 ± 2.9	−3.2 ± 3.6	−0.1 ± 1.8	<0.001	0.57	<0.001	<0.001
Pattern standard deviation, dB	4.85 ± 4.74	5.13 ± 4.44	2.30 ± 1.02	<0.001	0.12	<0.001	<0.001
VF index, %	91.9 ± 5.1	88.9 ± 8.7	98.9 ± 1.4	<0.001	0.12	<0.001	<0.001

BCVA: best-corrected visual acuity; IOP: intraocular pressure; NTG-SRD: normal tension glaucoma with superior retinal nerve fiber layer defect; SE: spherical equivalent; SSOH: superior segmental optic hypoplasia; VF: visual field.

**Table 2:** Comparison of optical coherence tomography parameters as determined by cirrus high-definition optical coherence tomography.

	SSOH (A)	NTG-SRD (B)	Controls (C)	*P* value	*P* value		
					Post hoc comparison		
					A-B	A-C	B-C
pRNFL thickness, *µ*m							
Average	73.0 (10.3)	73.1 (6.8)	92.1 (6.2)	<0.001	0.99	<0.001	<0.001
Temporal	64.4 (13.0)	54.1 (13.1)	70.3 (14.5)	<0.001	0.06	0.289	<0.001
Superior	72.8 (12.8)	75.0 (13.2)	115.3 (15.9)	<0.001	0.89	<0.001	<0.001
Nasal	58.1 (8.6)	63.6 (7.1)	70.1 (9.3)	<0.001	0.13	<0.001	0.004
Inferior	96.1 (19.5)	98.6 (14.9)	116.2 (15.3)	<0.001	0.88	0.001	0.002
1 o/c	65.1 (18.3)	83.8 (22.3)	107.9 (20.5)	<0.001	0.02	<0.001	0.006
2 o/c	63.2 (13.1)	73.5 (15.7)	80.6 (13.5)	0.047	0.04	0.049	0.299
3 o/c	54.7 (10.9)	57.7 (9.1)	62.5 (9.35)	0.043	0.06	<0.001	0.991
4 o/c	56.8 (9.7)	59.4 (9.6)	64.7 (10.2)	<0.001	0.71	0.001	0.060
5 o/c	70.4 (13.8)	83.6 (16.1)	89.0 (20.1)	0.003	0.06	0.002	0.430
6 o/c	106.9 (34.9)	106.4 (20.37)	120.9 (28.3)	0.226			
7 o/c	119.5 (29.2)	108.7 (26.4)	136.1 (25.8)	0.009	0.43	0.26	0.006
8 o/c	71.7 (15.6)	63.7 (19.1)	74.1 (18.1)	0.060	0.37	0.73	0.048
9 o/c	53.6 (12.2)	46.3 (10.0)	57.0 (13.1)	0.002	0.15	0.36	0.010
10 o/c	69.8 (19.6)	53.0 (15.2)	80.2 (17.4)	<0.001	0.01	0.048	<0.001
11 o/c	85.1 (24.7)	63.3 (15.8)	122.8 (24.8)	<0.001	0.01	<0.001	<0.001
12 o/c	66.5 (11.9)	77.7 (19.8)	115.2 (24.4)	<0.001	0.22	<0.001	<0.001
GCIPL thickness, *µ*m							
Average	73.2 (6.1)	72.2 (7.4)	81.5 (10.7)	<0.001	0.94	0.002	<0.001
Minimum	66.8 (8.7)	60.9 (10.0)	79.2 (7.0)	<0.001	0.09	<0.001	<0.001
Superotemporal	69.6 (7.4)	63.5 (10.4)	80.4 (13.8)	<0.001	0.23	0.003	<0.001
Superior	70.7 (7.3)	69.5 (11.4)	81.8 (12.3)	<0.001	0.95	0.001	<0.001
Superonasal	81.9 (7.0)	74.1 (10.1)	83.6 (11.4)	<0.001	0.03	0.160	<0.001
Inferotemporal	71.8 (10.2)	75.2 (9.6)	81.4 (13.9)	0.009	0.65	0.011	0.046
Inferior	72.1 (8.5)	74.6 (7.5)	80.0 (7.0)	0.001	0.55	0.001	0.014
Inferonasal	78.9 (8.1)	76.4 (6.7)	81.5 (8.9)	0.013	0.63	0.197	0.011

GCIPL: ganglion cell inner plexiform layer; NTG-SRD: normal tension glaucoma with superior retinal nerve fiber layer defect; pRNFL: peripapillary retinal nerve fiber layer; SSOH: superior segmental optic hypoplasia.
